# Maternal Baicalin Treatment Increases Fetal Lung Surfactant Phospholipids in Rats

**DOI:** 10.1093/ecam/nep073

**Published:** 2011-01-04

**Authors:** Chung-Ming Chen, Leng-Fang Wang, Kur-Ta Cheng

**Affiliations:** ^1^Department of Pediatrics, Taipei Medical University Hospital, Taipei, Taiwan; ^2^Department of Pediatrics, School of Medicine, College of Medicine, Taipei Medical University, Taipei, Taiwan; ^3^Department of Biochemistry, School of Medicine, College of Medicine, Taipei Medical University, Taipei, Taiwan

## Abstract

Baicalin is a flavonoid compound purified from the medicinal plant *Scutellaria baicalensis* Georgi and has been reported to stimulate surfactant protein (SP)-A gene expression in human lung epithelial cell lines (H441). The aims of this study were to determine whether maternal baicalin treatment could increase lung surfactant production and induce lung maturation in fetal rats. This study was performed with timed pregnant Sprague-Dawley rats. One-day baicalin group mothers were injected intraperitoneally with baicalin (5 mg/kg/day) on Day 18 of gestation. Two-day baicalin group mothers were injected intraperitoneally with baicalin (5 mg/kg/day) on Days 17 and 18 of gestation. Control group mothers were injected with vehicle alone on Day 18 of gestation. On Day 19 of gestation, fetuses were delivered by cesarean section. Maternal treatment with 2-day baicalin significantly increased saturated phospholipid when compared with control group and total phospholipid in fetal lung tissue when compared with control and 1-day baicalin groups. Antenatal treatment with 2-day baicalin significantly increased maternal growth hormone when compared with control group. Fetal lung SP-A mRNA expression and maternal serum corticosterone levels were comparable among the three experimental groups. Maternal baicalin treatment increases pulmonary surfactant phospholipids of fetal rat lungs and the improvement was associated with increased maternal serum growth hormone. These results suggest that antenatal baicalin treatment might accelerate fetal rat lung maturation.

## 1. Introduction

Respiratory distress syndrome (RDS) is a major cause of morbidity and mortality in preterm neonates. Maternal glucocorticoid treatments given to women at high risk of preterm delivery have been used extensively to decrease the incidence and severity of RDS [1]. It was suggested that the beneficial effect of glucocorticoid did not occur if there was an interval of over 7 days between treatment and delivery [2]. These findings persuaded obstetricians to repeat the course of glucocorticoid after 7 days in pregnant women at risk of preterm delivery who had not yet given birth. However, there is considerable evidence from laboratory and clinical studies that repeated antenatal steroid administration is associated with decreased birth body weight, length and head circumference and no additional benefits in preterm-birth outcomes [3, 4]. These studies indicate that maternal glucocorticoid could not completely prevent the incidence of RDS and have untoward neurological side effects after multiple doses and that there are other potent lung maturation factors that remain to be identified.

**Figure 1:**
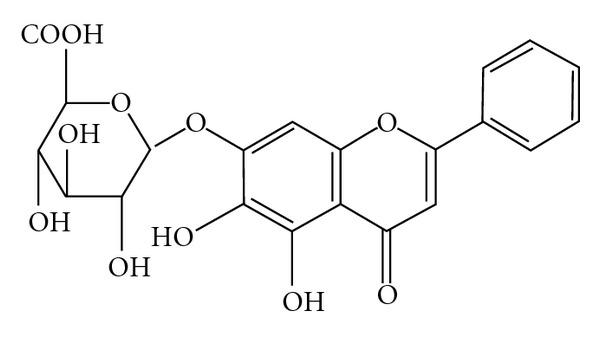
Chemical structure of baicalin.

The extract from the root of *Scutellaria baicalensis* Georgi (Scutellaria Radix) is the main component in Chinese medicine prescribed for miscarriage, threatened abortion, and neonatal hyperbilirubinemia. It is composed of four major flavenoids, that is, baicalin, baicalein, wogonin and wogonoside. Baicalin (Figure 1), 5,6,7-trihydroxyflavone-7-*β*-D-glucuronide, has antibacterial, antiviral, anti-inflammatory, anti-oxidative and anticancer activities [5–8]. It has been used as an anti-inflammatory and protective agents in the treatment of experimental pancreatitis and liver injury [9, 10]. We found that baicalin stimulates surfactant protein (SP)-A gene expression through induction of cytochrome c oxidase in human lung epithelial cell lines (H441) [11]. We hypothesized that maternal baicalin treatment might increase fetal lung surfactant production *in vivo* in preterm rats and quantified the effects by biochemical and molecular analyses.

## 2. Methods

### 2.1. Animals and Experimental Procedures

The Animal Care and Use Committee at Taipei Medical University approved this study. This study was performed with timed pregnant Sprague-Dawley rats (vaginal smear positive, Day 0; term, Day 22). One-day baicalin group mothers (*n* = 2) were injected intraperitoneally (i.p.) with baicalin (5 mg/kg/day, Wako Pure Chemical Industries, Japan) on Day 18 of gestation. Two-day baicalin group mothers (*n* = 3) were injected i.p. with baicalin (5 mg/kg/day) on Days 17 and 18 of gestation. Baicalin was dissolved in dimethyl sulfoxide (DMSO) at a concentration of 5 mg/ml. The dose of baicalin was based on the work of P.-L. Tsai and T.-H. Tsai [12]. Control group mothers (*n* = 3) were similarly injected with DMSO alone on Day 18 of gestation. On Day 19 of gestation, all dams were anesthetized with pentobarbital (50 mg/kg, i.p., Abbott Laboratories, North Chicago, IL, USA), and cesarean section was used to deliver the fetuses. At delivery, the fetuses were weighed and killed by an i.p. injection of pentobarbital (100 mg/kg). The organs of interest were then dissected free and weighed to the nearest 0.1 mg. Results were expressed as organ weight and the ratio (%) of organ/body weight. After delivery of the fetuses, a blood sample was taken from the pregnant mother before she was sacrificed. Plasma was immediately separated from blood cells by centrifugation and kept at –20°C for hormone measurements.

### 2.2. Biochemical Analysis

Lungs were homogenized and aliquots were extracted with chloroform-methanol [13]. Lipid extracts from aliquots of the lung homogenates were treated with osmium tetroxide, and saturated phospholipid was recovered by alumina column chromatography and quantified using a phosphorus assay [14, 15]. Values were expressed as *µ*mol/g protein. Aliquots of the lung homogenate from each animal were used to measure the total protein content by Bio-Rad protein assay kit using bovine serum albumin as a standard.

Maternal serum growth hormone concentrations were measured using a commercially available radioimmunoassay kit (Diagnostic Systems Laboratories, Webster, TX, USA). Maternal serum corticosterone levels were measured using an enzyme immunoassay kit (BIOCODE, Liège, Belgium).

### 2.3. Molecular Analysis

Gene expression of SP-A was measured with reverse transcriptase-polymerase chain reaction (RT-PCR). Total RNA was isolated from lung using the guanidine isothiocyanate based TRIzol solution (Invitrogen Life Technologies, Paisley, UK) according to the manufacturer's specifications and quantified by measurement of absorbance at 260 nm. Total RNA (2 *µ*g) was converted to first strand cDNA with oligo(dT) primer and reverse transcriptase. The primer sequences used in PCR for SP-A cDNA are 5′-GGAAGCCCTGGGATCCCTGGA-3′ and 5′-AGGCTTTGTCCCCACAG-3′ with an expected size of 558 base pairs (accession no. M33201). *β*-Actin (BD Biosciences Clontech, Palo Alto, CA, USA) was used as an internal control. The PCR reactions were carried out using the following conditions: 94°C for 1 min followed by 60°C for 1 min and 72°C for 1 min. The primer annealing temperature was 60°C with 29 cycles for SP-A and 33 cycles for *β*-actin. SP-A mRNA expressions were determined by running the samples at the optimal cycle number, which was selected in the region of linearity between cycle number and PCR product intensity. Products were visualized on ethidium bromide-stained gels and quantified using the BioRad Gel Documentation System (BioRad Laboratories, Hercules, CA, USA).

### 2.4. Statistical Analysis

Results are presented as the mean ± SEM. Statistically significant differences were analyzed by ANOVA followed by Bonferroni post hoc analysis. Differences were considered significant at *P* < .05.

## 3. Results

There were 27 fetuses from three rats in the control group, 21 fetuses from two rats in the 1-day baicalin group and 22 fetuses from three rats in the 2-day baicalin group. There was no significant difference in litter size among the three experimental groups.

### 3.1. Maternal Baicalin Treatment Effects on Fetal Body Weight, Organ Weight and Organ/Body Weight Ratio (%)

Effects of maternal baicalin treatment on fetal body weight and the organ/body weight ratio (%) are presented in Tables 1 and 2. The body weight, lung, brain and kidney weights, and lung/body weight, brain/body weight and kidney/body weight ratios were comparable among the three experimental groups. Two-day baicalin-treated fetuses had significantly higher liver weight and liver/body weight ratio than did the control group.

### 3.2. Fetal Lung Phospholipid

Maternal treatment with baicalin increased saturated phospholipid in fetal lung tissue, and the value reached statistical significance in the 2-day baicalin-treated group when compared with control animals (Table 3). Two-day baicalin-treated fetuses had significantly higher total phospholipid than did the control and 1-day baicalin-treated groups.

### 3.3. Fetal Lung SP-A mRNA Expression

**Figure 2:**
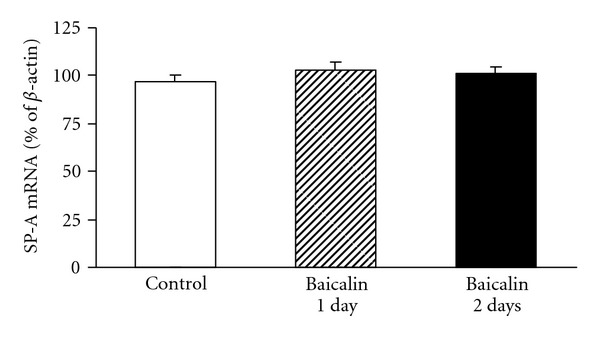
Maternal baicalin treatment effects on SP-A mRNA expression of fetal lung tissue in the control (*n* = 23), 1-day baicalin (*n* = 19)- and 2-day baicalin (*n* = 15)-treated preterm rats. Data are expressed as the mean ± SEM.

The SP-A mRNA expression in fetal lung tissue was comparable among the three experimental groups (Figure 2).

### 3.4. Growth Hormone and Corticosterone Levels in Maternal Serum at Delivery

**Figure 3:**
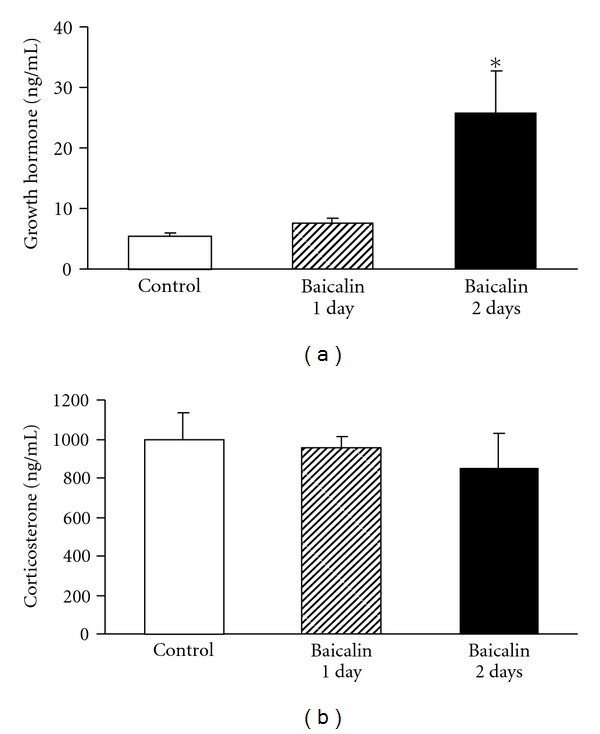
(a) Growth hormone and (b) corticosterone serum levels in the control (*n* = 3), 1-day baicalin (*n* = 2)- and 2-day baicalin (*n* = 3)-treated dams. Data are expressed as the mean ± SEM. *P < .05 compared with the control group.

Antenatal treatment with 2-day baicalin significantly increased maternal growth hormone when compared with control group (Figure 3(a)). Corticosterone concentrations in maternal serum were comparable among the three experimental groups (Figure 3(b)).

## 4. Discussion

Neonatal respiratory failure is a serious clinical problem associated with high morbidity, mortality and costs [16, 17]. The major risk factor is premature birth and its associated RDS. The pathophysiology of RDS is an immature lung structure and a deficit of pulmonary surfactant. Glucocorticoids have been reported to enhance fetal lung maturation and surfactant production [18]. However, there is considerable evidence that glucocorticoids have an adverse effect on the growth and development of the immature brain [3, 4]. Baicalin has been reported to stimulate SP-A gene expression in vitro after 48 h of incubation [11]. Therefore, we investigated the effects of antenatal baicalin treatment 48 h before birth in preterm rats that have been shown to be a suitable model for the study of acute neonatal lung disease [19].

In this study, we found that antenatal baicalin treatment did not influence fetal body, brain, lung and kidney weight and 2-day baicalin treatment significantly increased liver weight and liver/body weight ratio as compared with that of the control group. It appears that baicalin promotes liver growth in this premature animal model. The increase in liver weight in the offspring of baicalin-treated dams is of interest, as others have reported that baicalin could prevent experimental hepatic fibrosis [20], although, to our knowledge, no increase in liver weight has been recorded.

Pulmonary surfactant stabilizes the lung by producing a surface-active monolayer that reduces the surface tension at the air-liquid interface of the terminal airways. This reduction in surface tension contributes to mechanical stability by preventing alveolar and bronchiolar collapse during expiration. Surfactant is composed of *∼*90% lipids and 10% proteins. The surface activity property is due primarily to dipalmitoylphosphatidylcholine, which is *∼*50% of surfactant by weight. Therefore, we measured the saturated phospholipid content in fetal lung tissue in this premature animal model. Four lung-specific proteins (SP-A, SP-B, SP-C and SP-D) have been found to be associated with the surfactant [21]. Surfactant proteins are required both for the transition between lamellar bodies and tubular myelin, and for the spreading of tubular myelin components to the surface film. SP-A, the major pulmonary surfactant-associated protein, is a developmentally and hormonally regulated sialoglycoprotein of about 35 000 mol wt. constituting about 50% of the total surfactant protein [22]. In addition to its direct effects on film adsorption and stability, SP-A seems to enhance the resistance of surfactant to protein inhibition and stimulates phagocytosis of bacteria and viruses by alveolar macrophages [23]. In this study, we found that antenatal baicalin treatment increased total phospholipids and saturated phospholipid levels, and did not affect SP-A mRNA expression in fetal lung tissue. This result is in contrast to our previous report of increased SP-A gene expression after incubation of H441 cells in the presence of baicalin [11]. These differences may suggest that a higher level of baicalin stimulation is required to enhance *in vivo* surfactant protein gene expression or cell-cell and matrix-cell interactions might be implicated in the modulation of baicalin effects in lung development.

In this study, we found that 2-day baicalin treatment significantly increased maternal growth hormone levels. Growth hormone appears to have important effects on fetal metabolism and development. Lung is established as a target site for pituitary growth hormone action as pathophysiological states of pituitary growth hormone excess and deficiency are associated with impaired pulmonary function [24, 25]. Growth hormone may be involved in lung development because it is present in extra-pituitary tissues of rat embryos [26]. The growth hormone receptor gene is also expressed in pulmonary tissues [27, 28]. Therefore, pituitary growth hormone is possible to be an endocrine regulator of lung growth. These results suggest that baicalin has some hormonal effects. However, maternal corticosterone levels were comparable in the control and baicalin-treated groups. This result indicates that baicalin has no inhibitory effect on hypothalamic-pituitary-adrenal axis and the lung maturational effects of baicalin occurred independently of corticosterone [29].

**Figure 4:**
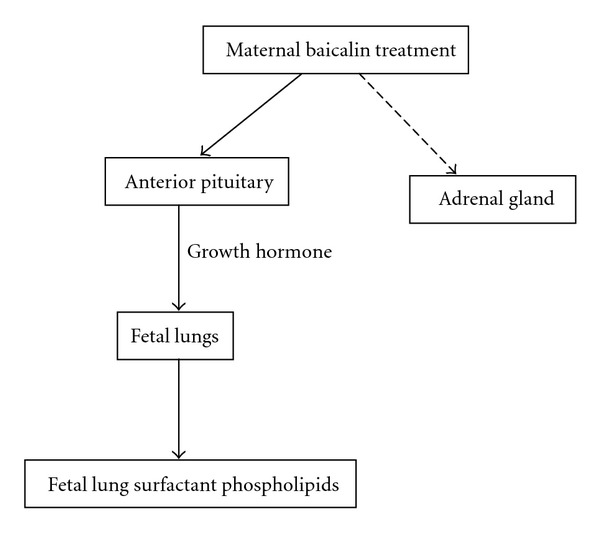
A hypothetical scheme of maternal baicalin treatment increases fetal rat lung surfactant phospholipids. Solid arrow indicates stimulation and dashed arrow indicates no effect.

In conclusion, maternal baicalin treatment at a dose of 5 mg/kg/day on Days 17 and 18 of gestation increases fetal rat lung surfactant phospholipids and the improvement was associated with increased maternal serum growth hormone.  Figure 4 is a hypothetical scheme of maternal baicalin treatment increases fetal rat lung surfactant phospholipids. These results suggest that antenatal baicalin treatment might accelerate fetal rat lung maturation.

## Funding

National Science Council (NSC90-2314-B-038-011).

## Figures and Tables

**Table 1:** Maternal treatment effects on fetal body weight and lung/body weight ratio.

Treatment	*n*	Body weight (g)	Lung weight (g)	Lung/body weight (%)
Control	27	2.48 ± 0.07	0.098 ± 0.004	3.90 ± 0.09
Baicalin (1 day)	21	2.62 ± 0.05	0.094 ± 0.002	3.63 ± 0.08
Baicalin (2 days)	22	2.46 ± 0.05	0.092 ± 0.002	3.80 ± 0.07

Values are mean ± SEM. *n* is the number of fetuses tested.

**Table 2:** Maternal treatment effects on organ weight and organ/body weight ratio.

Treatment	*n*	Brain weight (g)	Brain/body weight (%)	Kidney weight (g)	Kidney/body weight (%)	Liver weight (g)	Liver/body weight (%)
Control	27	0.109 ± 0.003	4.47 ± 0.12	0.020 ± 0.001	0.81 ± 0.02	0.22 ± 0.01	8.67 ± 0.21
Baicalin (1 day)	21	0.113 ± 0.002	4.37 ± 0.08	0.020 ± 0.000	0.79 ± 0.02	0.23 ± 0.00	8.97 ± 0.17
Baicalin (2 days)	22	0.113 ± 0.04	4.32 ± 0.15	0.021 ± 0.001	0.81 ± 0.02	0.25 ± 0.01*	9.54 ± 1.01**

Values are mean ± SEM. *n* is the number of fetuses tested. *P < .05, **P < .01 compared with the control group.

**Table 3:** Maternal treatment effects on saturated phosphatidylcholine and total phospholipids in fetal lung tissue of preterm rats.

Treatment	*n*	Saturated phospholipid (*μ*mol/g protein)	Total phospholipid (*μ*mol/g protein)
Control	9	31.02 ± 0.98	199.21 ± 9.57
Baicalin (1 day)	6	36.09 ± 1.45	216.24 ± 4.67
Baicalin (2 days)	13	39.35 ± 1.97^#^	636.01 ± 92.20**

Values are mean ± SEM. *n* is the number of fetuses tested. **P < .01 compared with the control and 1-day baicalin groups, ^#^ *P* < .01 compared with the control group.
